# Rolling Bearing Fault Diagnosis Based on Refined Composite Multi-Scale Approximate Entropy and Optimized Probabilistic Neural Network

**DOI:** 10.3390/e23020259

**Published:** 2021-02-23

**Authors:** Jianpeng Ma, Zhenghui Li, Chengwei Li, Liwei Zhan, Guang-Zhu Zhang

**Affiliations:** 1 School of Instrumentation Science and Engineering, Harbin Institute of Technology, Harbin 150001, China; 18B901017@stu.hit.edu.cn; 2 Aero Engine Corporation of China Harbin Bearing Co., LTD, Harbin 150500, China; zhenghuili90@163.com (Z.L.); zhanliwei333@163.com (L.Z.); 3 Songsin Global Campus, Undergraduate College, The Catholic University of Korea, Bucheon-Si, Gyeonggi-do 14662, Korea; zhangks@catholic.ac.kr

**Keywords:** refined composite multi-scale approximate entropy, coyote optimized algorithm, probabilistic neural network, rolling bearing, fault diagnosis

## Abstract

A rolling bearing early fault diagnosis method is proposed in this paper, which is derived from a refined composite multi-scale approximate entropy (RCMAE) and improved coyote optimization algorithm based probabilistic neural network (ICOA-PNN) algorithm. Rolling bearing early fault diagnosis is a time-sensitive task, which is significant to ensure the reliability and safety of mechanical fault system. At the same time, the early fault features are masked by strong background noise, which also brings difficulties to fault diagnosis. So, we firstly utilize the composite ensemble intrinsic time-scale decomposition with adaptive noise method (CEITDAN) to decompose the signal at different scales, and then the refined composite multi-scale approximate entropy of the first signal component is calculated to analyze the complexity of describing the vibration signal. Afterwards, in order to obtain higher recognition accuracy, the improved coyote optimization algorithm based probabilistic neural network classifiers is employed for pattern recognition. Finally, the feasibility and effectiveness of this method are verified by rolling bearing early fault diagnosis experiment.

## 1. Introduction

Rolling bearings are common connecting and fixing parts in rotating machinery, which have the advantages of high running precision, good substitutability, low price and scale production [1]. However, due to the influence of alternating load, machining error, improper installation and other factors, the rolling bearing will be damaged during the working process. The rotating machine will not work properly, and a catastrophic accident may even occur [2]. Furthermore, the vibration signals of the rolling bearings are usually nonlinear and non-stationary with the existence of various nonlinear factors (such as material strength and skid friction), and early faults are always submerged by strong background noise, which could increase the difficulty of fault diagnosis [3]. Therefore, it has become an urgent problem to find effective methods of rolling bearing early fault feature extraction and pattern recognition. In recent years, the fault diagnosis method based on machine learning has attracted much attention in the field of rolling bearing early fault diagnosis. These methods mainly include three steps: feature extraction, descent and pattern recognition [4,5]. With the development of nonlinear technology, many nonlinear dynamic methods based on statistical parameter estimation have been applied to extract fault features [6,7,8,9]. The most popular technique are correlation dimension and entropy-based measurement. Nevertheless, reliable estimation of correlation dimensions requires a long-term time series, which brings great limitations to the analysis of short-term vibration signals. Entropy-based measures include sample entropy, fuzzy entropy, permutation entropy, etc. The initial entropy-based measurement only completes a single-scale analysis, which typically assigns the highest value to highly unpredictable random signals rather than structurally complex signals. Hence, the single-scale entropy metric could not physically quantify the complexity of the time series [10]. Costa et al. proposed multi-scale entropy (MSE) algorithm in [10,11] and applied it to rolling bearing fault diagnosis for the first time in [12]. According to the MSE algorithm, the original time series is initially divided into non-overlapping segments with a length of (called proportional factor). Next, the time series of coarse granularity is obtained by calculating the average value of each fragment. Finally, the sample entropy of coarse-grained time series at each scale is calculated. The application of mean square error in feature extraction of mechanical vibration signal is also successful. The mean square error and adaptive neuro fuzzy inference is employed to detect rolling bearing faults and determine their severity [13]. Hsieh et al. utilized the mean square error curve to identify some characteristic defect of the high-speed spindle [14]. The traditional MSE algorithms will shorten the data set and produce uncertain short-term data values when large scale factors are utilized. To make up for these shortcomings, Wu et al. proposed an improved multi-scale entropy to obtain more template vectors [15]. However, the improved multiscale algorithm greatly increases the computation time. Later, composite multiscale sample entropy (CMSE) [16] and refined composite multi-scale sample entropy (RCMSE) [17] are developed for novel coarse-grained processes. Wang et al. proposed a modified multiscale weighted permutation entropy for rolling bearing fault diagnosis [18].

It is necessary to employ a classifier when performing pattern recognition in a low-dimensional feature set. Many classifiers have been proposed and applied to the fault detection process in rotating machinery, such as expert system [19], artificial neural network [20], and fuzzy logic classifier [21]. However, these classifiers have some drawbacks (e.g., local optimal solution, low convergence rate and significant overfitting). The above contents limit their application in pattern recognition rolling bearing fault detection. Probabilistic neural network (PNN) is a supervised neural network that is commonly used in pattern recognition [22]. Because of its parallel distributed processing, self-learning and self-organization, the PNN model has good application potential in fault diagnosis. Compared with traditional neural network learning methods, the PNN model learning process mainly employs Parzen nonparametric probability density function estimation [23] and Bayes classification rules [24]. The PNN model will converge to a Bayes classifier if there are enough training samples.

Machine learning is a growing field that attempts to extract knowledge from data sets. It is usually in the form of algorithms that predict results. The tasks of machine learning include classification, regression, clustering, time series prediction and so on. In the classification task, the ideal prediction result is the class of each instance in the dataset. In general, the classifier goes through at least two stages: training and validation. The coyote optimization algorithm (COA), which was introduced by Pierezan and Coelho, is mainly inspired by the coyotes living in North America [25]. The COA finds a solution to the optimization problem by learning from the social organization of coyotes and their adaptation to the environment. The COA is a population-based algorithm divided into population intelligence and evolutionary heuristics. Furthermore, it has different algorithmic structures that focus on social structures and coyote-communicated experiences rather than just catching prey, similar to other AI algorithms such as grey wolf optimizer [26]. In [25], it suggests that intrinsic factors (gender, social status, and populations to which coyotes belong) and extrinsic factors (e.g., snow depth, snow hardness, temperature, and cadaver biomass) influence coyote activity. Hence, the COA mechanism is designed according to the social conditions of coyotes, which means the decision variables of the global optimization problem. From an optimization perspective, each coyote corresponds to a feasible solution. The quality of each coyote’s social conditions the result of the application of the objective function in the social condition. The optimal social condition is the global solution of the problem.

In contrast to existing researches, a rolling bearing early fault diagnosis model based on complete ensemble intrinsic time-scale decomposition with adaptive noise (CEITDAN), refined composite multi-scale approximate entropy (RCMAE) and improved coyote optimization algorithm based probabilistic neural network (ICOA-PNN) is proposed in this paper. The RCMAE proposed in the paper could reduce the possibility of inducing uncertain entropy and is well suitable for bearing early fault diagnosis. In the improved coyote optimization algorithm, we employed a differential evolution algorithm instead of the traditional greedy iterative algorithm and utilized the dynamic adjustment of the coyote method to optimize the process of coyote removal and acceptance, which improves the optimization effect of coyote optimization algorithm. The model consists of three steps (feature extraction, descent and pattern recognition). Firstly, the original signal is decomposed by CEITDAN, and the RCMAE, approximate period and approximate energy are calculated and constructed to construct the original three-dimensional collection. Finally, this three-dimensional collection automatically identifies fault types as input for ICOA-PNN machine learning. The fault diagnosis experiment of the rolling bearing shows that the proposed method has a higher recognition accuracy for bearing conditions under various working conditions.

The structure of the paper is as follows: Section 2 introduces the principle of CEITDAN algorithm. Section 3 introduces the RCMAE, approximate period and approximate energy, and verifies the effectiveness of the algorithm by noise signal analysis experiment. In Section 4 and Section 5, a new fault diagnosis method of rolling bearing is proposed. Section 6 gives the experimental evaluation. We conclude in Section 7.

## 2. CEITDAN—Based Signal Decomposition

In order to pre-process the noisy signal, this article uses the CEITDAN method to decompose the signal [27]. The residual component is obtained, white noise is added to the residual component, and the same operation is performed, until the residual component is a monotonic function or the extreme points are less than three. The proper rotation component can accurately define the instantaneous information of the signal and is repeated several times in this manner.

The core idea of CEITDAN method denoising is that by adding a group of white noise to the original signal, the added white noise can be adaptively decomposed together with the noise part of the original signal during the decomposition process. Firstly, proper rotation components (PRCs) of white noise preprocessed by intrinsic time-scale decomposition (ITD) are added in different decomposition stages. This process can help ITD to establish a global scale reference. Then, ITD is used to decompose the noise-added input signal into a PRC and a residual. Theoretically, according to the filter structure of ITD, most of the added noise and the signal components approximately proportional to the added noise are extracted into PRCs. Therefore, there is almost no added noise in the residual component. CEITDAN calculates the final PRC as the difference between the signal to be decomposed and the average value of the residual obtained by decomposition. Therefore, compared with ITD and complete ensemble empirical mode decomposition with adaptive noise (CEEMDAN), PRC extracted by CEITDAN has a more appropriate scale and contains less residual noise. In addition, due to the pretreatment of ITD, the increased noise does not include the local average. This can reduce the number of filtering iterations in each stage.

Step 1. First round of noise addition: $\omega_{n}^{i}\left(t\right)\left(i=1,2,\cdots ,I\right)$ is the first order proper rotation component decomposed by ITD which from white noise with a certain SNR, where I is the number of noise additions, is superimposed onto the original signal x(t). As the average value is used as the residue component of the method, then, the first-order component of the origin signal is obtained as follows:

(1) $$r_{1}\left(t\right)=\frac{1}{I}\sum_{i=1}^{I}L_{1}\left[\beta_{0}\omega_{n}^{i}\left(t\right)+x\left(t\right)\right]$$

n is the corresponding order of ITD decomposition white noise.$\beta_{0}$ is used as $\beta_{0}=\varepsilon_{0}std\left(x\right)/std\left(\omega^{\left(i\right)}\right)$ and $\varepsilon_{0}$ the noise-adding amplitude coefficient, $L_{k}\left[A\left(t\right)\right]$ represents the residue component obtained by ITD decomposing signal A(t). A(t) is the signal with white noise. The proper component at this time are as follows:

(2) $$PR_{1}=x\left(t\right)-r_{1}\left(t\right)$$

Step 2. Second round of noise addition: The second-order PR components of white noise $\omega^{i}\left(t\right)$ are superimposed onto $r_{1}\left(t\right)$, and the first-order component of the mixed signal are obtained by ITD decomposition. The average is used as the second residue component of this method’s residue component, as follows:

(3) $$r_{2}\left(t\right)=\frac{1}{I}\sum_{i=1}^{I}L_{1}\left[\beta_{1}L_{1}\left(\omega^{i}\left(t\right)\right)+r_{1}\left(t\right)\right]$$

The second-order proper component obtained at this time are as follows:

(4) $$PR_{2}=r_{1}\left(t\right)-r_{2}\left(t\right)$$

Step 3. m-th round of noise addition: The (m−1)-th -order PR components of white noise $\omega^{i}\left(t\right)$ are superimposed onto the remaining terms $r_{m-1}\left(t\right)$. Then, the first-order component of the signal with adaptive white noise is obtained by ITD decomposition, and the average value is taken as the m−th-order residue component of the method, as follows:

(5) $$r_{m}\left(t\right)=\frac{1}{I}\sum_{i=1}^{I}L_{1}\left[\beta_{m-1}L_{m-1}\left(\omega^{i}\left(t\right)\right)+r_{m-1}\left(t\right)\right],\exists L_{m-1}\left[\varepsilon \omega^{i}\left(t\right)\right]$$

The remaining items obtained at this time are as follows:

(6) $$PR_{m}=r_{m-1}\left(t\right)-r_{m}\left(t\right)$$

Step 4. Repeat steps 1–3 several times until the residual component is a monotonic function or the number of extreme points in this residual component is fewer than three. The final remaining term is as follows:

(7) $$r_{M}\left(t\right)=r_{M-1}\left(t\right)-PR_{M}$$

At this point, the entire CEITDAN decomposition process ends.

The final shifted terms are as follows:

(8) $$x\left(t\right)=\sum_{m=1}^{M}PR_{m}+r^{M}\left(t\right)$$

Equation (8) elucidates the original signal x(t) as the sum of a series of PR components and the remainder; thus, it completes the CEITDAN method. The error of reconstructing the original signal by its decomposition result is theoretically zero.

**Figure 1:**
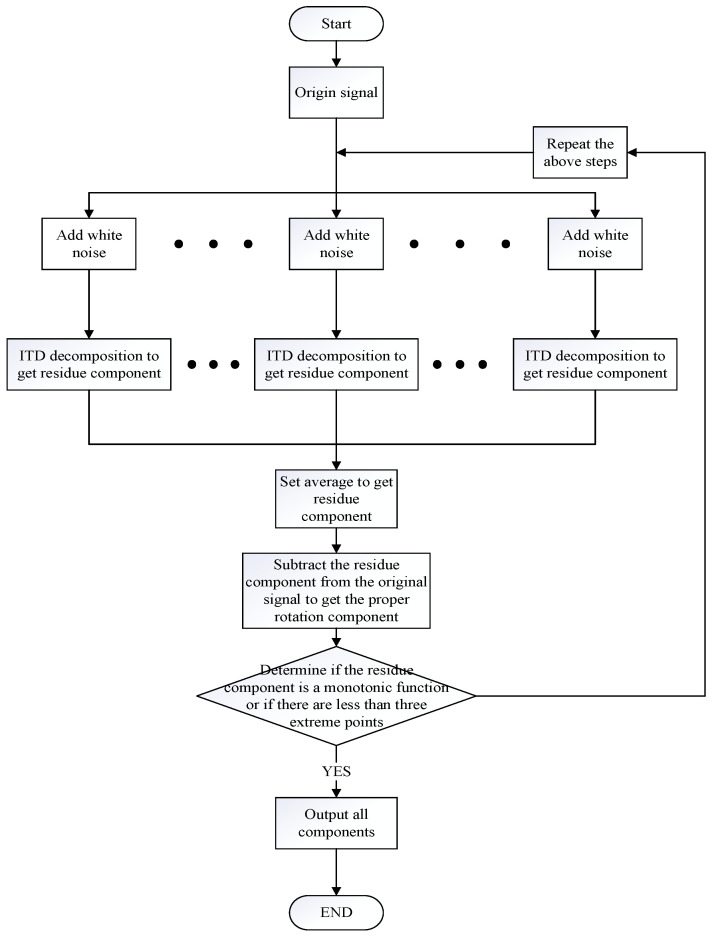
Composite ensemble intrinsic time-scale decomposition with adaptive noise method (CEITDAN) decomposition steps.

The decomposition steps of the proposed method are shown in Figure 1.

## 3. Feature Extraction Based on Refined Composite Multi-Scale Approximate Entropy and Approximate Period and Approximate Energy

Approximate entropy is a method to measure the complexity of time series, which has the advantages of strong anti-interference ability and short data required [28]. Approximate entropy could measure the complexity of a time series on a single scale, while multi-scale entropy could measure the complexity of a time series and detect small changes effectively. If the rolling bearing fails, the nonlinear dynamic complexity will also change. The mean square error is very suitable for feature extraction in the case of rolling bearing failure. However, when the coarse-grained process is employed to estimate the mean of each fragment, the dynamic mutation behavior of the time series is neutralized. Therefore, the calculated mean square error entropy is biased. The RCMAE algorithm is proposed to overcome this shortcoming. Meanwhile, in order to improve the accuracy of the bearing fault diagnosis, the RCMAE is extracted with approximate energy and approximate period [29] as characteristic parameters, and use approximate energy and approximate period to improve the accuracy of fault diagnosis.

### 3.1. Approximate Entropy and Multi-Scale Approximate Entropy

There is a known time series {x(1),x(2),⋯,x(N)} containing N points. The approximate entropy method is as follows.

(1) The pattern dimension is determined as m, and the phase space is reconstructed. The elements in the time series are extracted sequentially to form a vector sequence with m dimension.
  (9) X(i)={x(i),x(i+1),⋯,x(i+m−1)},i=1,2,⋯,N−m+1
(2) The distance between vector X(i) and X(j) is d[X(i),X(j)], which defined as the absolute value of the maximum difference between the two corresponding elements. That is,
  (10) d[X(i),X(j)]=max|x(i+k)−x(j+k)|,k=0,1,⋯,m−1;i,j=1,2,⋯,N−m+1
(3) The threshold of similar tolerance r is given. Define the values in d[X(i),X(j)] less than r as n, and calculate its ratio to the number of vectors.
  (11) $$C_{i}^{m}\left(r\right)=\frac{n}{N-m+1},i,j=1,2,\cdots ,N-m+1,i\neq j$$
(4) Define $\Phi^{m}\left(r\right)$ as the self-correlation of sequence:
  (12) $$\left\{X_{i}\right\}\Phi^{m}\left(r\right)=\frac{1}{N-m+1}\sum_{i=1}^{N-m+1}\ln C_{i}^{m}\left(r\right)$$
(5) Add 1 to the pattern dimension m and repeat above steps, we can get $\Phi^{m+1}\left(r\right)$.
(6) Define ApEn as the approximate entropy of the time series, then:
  (13) $$ApEn\left(m,r\right)=\Phi^{m}\left(r\right)-\Phi^{m+1}\left(r\right)$$

The multi-scale approximate entropy is the approximate entropy at different scales. The calculation process is as follows [10]:

(1) The coarse-grained data sequence $\left\{y_{j}^{\left(s\right)}\right\}$ is obtained by coarse-grained processing of time sequence {x(i),i=1,2,⋯,N}.
  (14) $$y_{j}^{\left(s\right)}=\frac{1}{s}\sum_{i=\left(j-1\right)s+1}^{js}x_{i},j=1,2,\cdots ,N/s$$
  where s is a scale factor. The raw data sequence is changed into coarse grain sequence with the length of N/s under different s.
(2) Calculating the approximate entropy of coarse grain sequence at each scale, the change of approximate entropy of raw data at different s could be obtained.

### 3.2. Refined Composite Multi-Scale Approximate Entropy

The RCMAE algorithm consists of two main steps. The calculation steps are as follows:

(1) For raw data {x(i),i=1,2,⋯,N}, the k-th coarse-grained sequence $y_{k}^{\left(s\right)}=\left\{y_{k,1}^{\left(s\right)},y_{k,2}^{\left(s\right)},\cdots \right\}$ is given by the following formula:
  (15) $$y_{k,j}^{\left(s\right)}=\frac{1}{s}\sum_{i=k+\left(j-1\right)s}^{k+js-1}x_{i},j=1,2,\cdots ,N/s,j=1,2,\cdots ,k=1,2,\cdots ,s$$
(2) For each scale s, the entropy values of RCMAE are defined as follows.
  (16) $$E_{RCMAE}\left(X,m,n,s\right)={\bar{\Phi }}^{m}\left(r\right)-{\bar{\Phi }}^{m+1}\left(r\right)$$
  (17) $$\bar{\Phi }\left(r\right)=\frac{1}{s}\sum_{k=1}^{s}/\Phi_{k}^{\left(t\right)}$$
  where $\bar{\Phi }\left(r\right)$ is the average value of the self-correlation of coarse-grained data sequence $y_{k}^{\left(s\right)}=\left\{y_{k,1}^{\left(s\right)},y_{k,2}^{\left(s\right)},\cdots \right\}$.

### 3.3. Approximate Energy and Approximate Period

It can be seen from the original data that energy and period are important parameters of the signal. We propose a method to calculate approximate energy and approximate period. This is a nonlinear dimension reduction method. The approximate energy and the approximate period are the approximate energy calculation process as follows.

Take the first mode component to get its $l^{p}$ model as follows.

(18) $${\left|\left|s\right|\right|}_{l^{p}}={\left(\frac{1}{N}\sum_{i=1}^{N}s{\left(i\right)}^{p}\right)}^{\frac{1}{p}}$$

The calculation of approximate p period of sequence X is:

Step 1. Normalization.
  (19) $$X=\frac{x-\min\left(x\right)}{\max\left(x\right)-\min\left(x\right)}$$
Step 2. Find the p power of the sequence:
  (20) $$X^{p}=\left[X{\left(1\right)}^{p},\ldots ,X{\left(i\right)}^{p},\ldots ,X{\left(N\right)}^{p}\right]$$
Step 3. Calculate the autocorrelation coefficient:
  (21) $$pX_{xcorr}=xcorr\left(X^{p}\right)$$
  (22) $$X_{xcorr}=xcorr\left(X^{1}\right)$$
Step 4. Normalize $pX_{xcorr}$ and $X_{xcorr}$.
Step 5. Calculate the autocorrelation coefficient of $pX_{xcorr}$ and $X_{xcorr}$.
Step 6. A simplified pattern sequence is obtained by dividing $pX_{xcorr}$ and $X_{xcorr}$
  (23) $$X^{\prime }=\frac{pX_{xcorr}}{X_{xcorr}}$$
  Intercept the [−N,N] of sequence $X^{\prime }$.
Step 7. Calculate the number of approximate periods and define it as approximate p periods of a sequence. ${\left|\left|X\right|\right|}_{l^{2}}={\left(\frac{\sum_{i=1}^{n}X{\left(n\right)}^{2}}{n}\right)}^{\frac{1}{2}}$ represents the energy of the signal in a sense, and it could be utilized to measure the energy of vibration to a certain extent, that is approximate energy.

The reason for choosing approximate period and approximate energy is for more accurate fault diagnosis. According to literature [27], a single variable will cause misjudgment. Therefore, this paper chooses to add two parameters of approximate period and approximate energy based on RCMAE.

### 3.4. Experimental Analysis of Noise Signal

**Figure 2:**
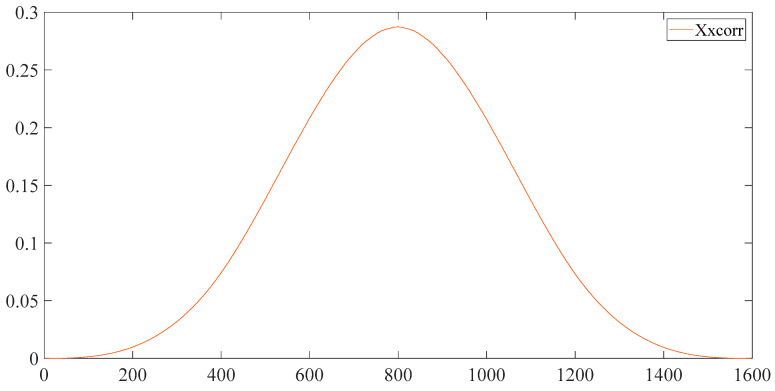
Autocorrelation coefficient of X.

Four parameters in the RCMAE need to be determined. They are embedded dimension m, respectively Signal length N, autocorrelation r and scale operator s. According to [17], m and s are set to 2 and 25 respectively. To make the approach more intuitive, we calculated autocorrelation coefficient and autocorrelation coefficient by employing a set of outer ring fault data from the West Reserve University bearing database, as the results shows in Figure 2.

When the original signal is impacted by abnormal signal, it will produce more energy signal locally. Abnormal signals are also periodic. Utilizing $X^{p}$, the larger part of the signal will be larger when p>1. The influence of the factor of period in the final sequence will be expanded by taking the correlation sequence. When the autocorrelation sequence of $X^{p}$ and $X^{1}$ is divided, the periodic parameters of our signal will be highlighted. Through the division of the autocorrelation coefficient of X and the correlation coefficient of $X^{p}$, the simplified pattern sequence could be obtained as follows.

**Figure 3:**
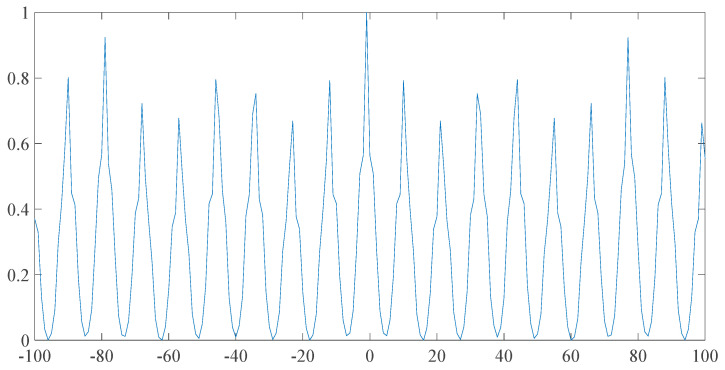
p-pattern sequence.

**Figure 4:**
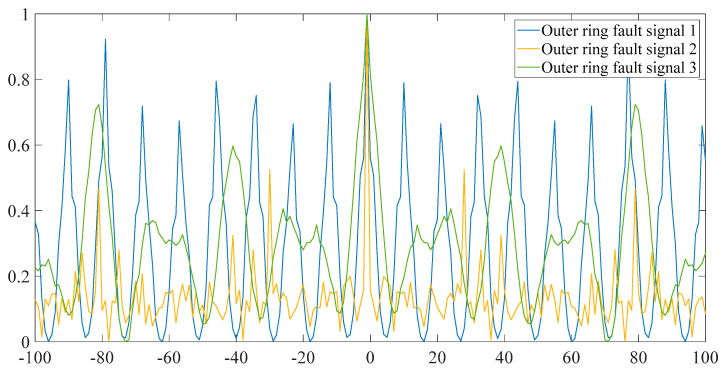
p-mode sequence of three abnormal signals.

As shown in Figure 3, this is a special observation. Our signal is greatly simplified, and the periodicity of the signal is obviously extracted. Two other sets of fault data with different outer ring and different crack size are employed to carry out the above simulation. Here is the p pattern sequence of the three fault signals in the following experimental data, as shown in Figure 4.

## 4. ICOA-PNN Pattern Recognition

In order to realize the intelligent fault diagnosis of rolling bearing, it is necessary to utilize classifier to identify the fault type. PNN classifier based on Bayesian strategy has good computational power and does not require backpropagation optimization parameters and training weights. It is applied to rolling bearing pattern recognition in this paper. There is an important parameter (i.e., the smoothing factor σ) that needs to be preset first before using the PNN. The smoothing factor σ greatly affects the recognition ability of probabilistic neural network models. Clearly, these two parameters have a great influence on PNN final pattern recognition results. To improve the PNN’s fault recognition ability, this section proposes an ICOA-PNN algorithm that uses the ICOA algorithm to determine the best parameters.

### 4.1. Coyote Optimization Algorithm

The COA, proposed by Pierezan et al. in 2018, is a new intelligent optimization algorithm that could simulate coyote social life, growth, death, group expulsion and acceptance [25]. COA divides the population into several subgroups through random grouping. It can be found that COA could achieve better optimization results in benchmark function optimization. By determining the coyote of the sub-group and cultural trends and randomly selecting two coyotes, these four factors will affect the growth of the coyotes, and we can then adjust the growth process based on the social adaptability of the coyotes. The birth of coyotes is affected by two randomly selected fathers and environmental variation. In terms of social adaptability, if the newborn coyote is better than the old and incompetent coyote, the old coyote dies; otherwise, the newborn coyote dies. Among the subgroups, according to a certain probability, some coyotes will be driven away by the group and accepted by other groups, thus changing the grouping state of the coyotes. Through the continuous evolution of the process of growth, death, expulsion, and acceptance, the coyote which is the most suitable for the social environment is obtained as the best solution to the optimization problem.

In COA, each coyote represents a candidate solution, and each solution vector is composed of coyote social state factors. These state factors include coyote internal and external factors, each state factor represents a decision variable, D state factors constitute a solution vector with D decision variables, and each coyote is measured by social adaptability. The COA is mainly divided into four stages: the random initialization and random grouping of suburban wolves, the growth of coyotes in the group, the life and death of the coyotes, and the group expulsion and acceptance of the coyotes.

(1) Initialize and group randomly. $lb_{j}$ Here, we set parameters such as the number of suburban wolves $N_{p}$, the number of suburban wolves in the group $N_{c}$, and the maximum number of iterations $N_{gen}$. The initial social state factors of each coyote are set immediately because COA is a random algorithm, as shown in Formula (24). The social adaptability of brown coyotes was calculated and randomly divided into groups:
  (24) $$soc_{j}=lb_{j}+r_{j}\times \left(ub_{j}-lb_{j}\right)$$
  where $lb_{j}$ and $ub_{j}$ denote the lower and upper bounds, respectively, of the j state factor of the coyotes; j=1,2,⋯,D; and $r_{j}$ is a random number with uniform distribution in [0,1].
(2) The growth of coyotes in the group. Here, we determine the optimal coyote alpha in the group, calculate the cultural trends of the group, randomly select two coyotes, and affect the growth of coyotes using these four factors. The calculation of the cultural trends of the group is shown in Formula (25):
  (25) $$cult_{j}=median(A_{j})$$
  where A is a matrix with $N_{c}$ rows and D rows and columns, which represents $N_{c}$ solution vectors; $A_{j}$ represents the A column of matrix j; and median represents the median. In the process of coyote growth, we first calculate the difference $\delta_{1}$ between the best coyote alpha in the group and one randomly selected coyote in the group, along with the cultural trend between the group and the other random coyote in group $\delta_{2}$, as shown in Formula (26). Then, the coyotes in the group grow under the influence of $\delta_{1}$ and $\delta_{2}$, as shown in Formula (27):
  (26) $$\delta_{1}=L_{best}-soc_{r1},\delta_{2}=cult-soc_{r2}$$
  where $r_{1}$ and $r_{2}$ represent two different random coyote markers, and $L_{bset}$ represents the best coyote alpha coyote in the group:
  (27) $$new\_soc_{c}=soc_{c}+s_{1}\times \delta_{1}+s_{2}\times \delta_{2}$$
  where $s_{1}$ and $s_{2}$ are random weights of $\delta_{1}$ and $\delta_{2}$, respectively; $s_{1}$ and $s_{2}$ are random numbers with uniform distribution in [0,1]. After each coyote in the group grows, the algorithm calculates the social adaptability and adopts greedy selection, as shown in Formula (28). By retaining the high-quality coyotes to participate in the growth of the other coyotes in the group, the convergence speed of the algorithm is accelerated:
  (28) $$soc_{c}=\{\begin{array}{c} new\_soc_{c},new\_fit_{c}<fit_{c}\\ soc_{c},otherwise \end{array}$$
(3) Life and death of coyotes. Two important evolution processes in nature are birth and death. In COA, the ages of the coyotes are measured in years. After each group of coyotes grows, a newborn coyote is born. The births and deaths of the coyotes are shown in Algorithm 1. The birth of new coyotes is influenced by the social conditions and social environments of two randomly selected parents. Newborn coyotes are produced in the manner shown in Formula (29):
  (29) $$pup_{j}=\{\begin{array}{c} soc_{j}^{cr1},rnd_{j}<P_{s}orj=j_{1}\\ soc_{j}^{cr2},rnd_{j}\geq P_{s}+P_{a}orj=j_{2}\\ R_{j},otherwise \end{array}$$
  where $cr_{1}$ and $cr_{2}$ are two randomly different coyotes in group p; $j_{1}$ and $j_{2}$ are two random dimensions of newborn coyotes; $P_{s}$ is the dispersion probability; and $P_{a}$ is the association probability, as shown in formula (30). Here, scattered association probability affects the diversity of newborn coyotes; $R_{j}$ is the random number of the j dimension of the decision variable; and $rnd_{j}$ is the random number with uniform distribution on [0,1],as shown in Algorithm 1:
  (30) $$P_{s}=\frac{1}{D},P_{a}=\frac{1-P_{s}}{2}$$
(4) Coyotes are driven away and accepted. At first, the coyotes are randomly assigned to each group, but some of the coyotes leave and join other groups. The probability of coyotes being expelled and accepted by the group is expressed by $P_{e}$, as shown in Formula (31). This mechanism facilitates the exchange of information among COA groups and promotes the interactions between coyotes among species:
  (31) $$P_{e}=0.005\times N_{c}^{2}$$

**Algorithm 1.** birth and death of coyotesStartCalculate ω and φIf φ=1Then, the newborn coyote survives, the only coyote in ω dies, and the age of the superior coyote is 0.If φ>1The newborn coyote survived, the oldest coyote with the worst social adaptability in ω died, and the age of the excellent coyote was 0OtherwiseThe newborn coyote diedEnd

After initialization and random grouping, the growth of coyotes, the life and death of coyotes, and the expulsion and acceptance of coyotes are carried out successively. If the iteration termination condition is reached, the optimal coyote is output; otherwise, jump to (3).

As can be seen from the above steps, COA has the following advantages: (a) COA has a better search model and framework. Coyotes are randomly divided into several sub-groups, and cultural communication is carried out through expulsion and acceptance after all groups of coyotes grow. Compared with algorithms such as PSO, this search model and framework has stronger exploration ability. (b) COA guides the growth of coyotes through wolves and cultural trends. This algorithm has strong local search ability. (c) COA. The generation of newborn coyotes emerges from the joint action of two randomly selected parents, the coyotes, and random mutations in the social environment, so the algorithm has certain global search ability. (d) The COA updates each coyote in the group. Compared with the particle swarm optimization (PSO) with a similar structure, the update method for COA is simple. (e) The COA was randomly grouped after initialization, and the coyotes in the group were randomly expelled and accepted, allowing the information between the groups to be exchanged.

COA shows strong optimization ability in the process of solving optimization problems. However, COA is a recent algorithm and needs to be improved and perfected. For example, there are the following problems in solving complex optimization problems: (a) The growth process of coyotes in COA affects the growth of coyotes by calculating the differences between the intra-group alpha coyote and the cultural trends and random selection of two coyotes in the group; moreover, the convergence speed of the algorithm is slow. (b) When guided by the intra-group alpha coyote and group culture trend, the intra-group alpha coyote and group culture trends may be the local optimal solution, leading to the local optimal of the algorithm. (c) The COA using the dynamic greedy algorithm accelerates the convergence speed to a certain extent but increases the probability of falling into the local optimal.

### 4.2. Probabilistic Neural Network (PNN)

The PNN algorithm belongs to a supervised learning pattern recognition algorithm in the field of machine learning. The PNN algorithm principle is mainly based on Bayesian minimum risk decision theory and artificial neural network (ANN) model. The probability density of sample population distribution is calculated by Parzen window estimation method to achieve the purpose of pattern classification. The learning process could be summarized as follows.

(1) The feature matrix of learning samples is normalized first and the number of training samples for each fault is set as p. The feature vector dimension of each sample is m and the input feature matrix is recorded as X.
  (32) $$X={\left[\begin{array}{cccc} x_{11} & x_{12} & \cdots & x_{1m}\\ x_{21} & x_{22} & \cdots & x_{2m}\\ \vdots & \vdots & \ddots & \vdots \\ x_{p1} & x_{p2} & \cdots & x_{pm} \end{array}\right]}_{p\times m}$$
  Calculate the module of each eigenvector in the input matrix and the matrix B is obtained.
  (33) $$B=\left[\begin{array}{cccc} 1/\sqrt{\sum_{k=1}^{n}x_{1k}^{2}} & 1/\sqrt{\sum_{k=1}^{n}x_{2k}^{2}} & \cdots & 1/\sqrt{\sum_{k=1}^{n}x_{pk}^{2}} \end{array}\right]$$
  Combine with (32) and (33), the normalized matrix C is obtained.
  (34) $$\begin{array}{l} C=B_{p1}{\left[\begin{array}{cccc} 1 & 1 & \cdots & 1 \end{array}\right]}_{1p}X_{pm}\\ =\left[\begin{array}{cccc} x_{11}/\sqrt{\sum_{k=1}^{n}x_{1k}^{2}} & x_{12}/\sqrt{\sum_{k=1}^{n}x_{1k}^{2}} & \cdots & x_{1m}/\sqrt{\sum_{k=1}^{n}x_{1k}^{2}}\\ x_{21}/\sqrt{\sum_{k=1}^{n}x_{2k}^{2}} & x_{22}/\sqrt{\sum_{k=1}^{n}x_{2k}^{2}} & \cdots & x_{2m}/\sqrt{\sum_{k=1}^{n}x_{2k}^{2}}\\ \vdots & \vdots & \ddots & \vdots \\ x_{p1}/\sqrt{\sum_{k=1}^{n}x_{pk}^{2}} & x_{p2}/\sqrt{\sum_{k=1}^{n}x_{pk}^{2}} & \cdots & x_{pm}/\sqrt{\sum_{k=1}^{n}x_{pk}^{2}} \end{array}\right] \end{array}$$
(2) The normalized sample data is input into the mode layer of probabilistic neural network. Assuming that the input sample matrix to be identified is p×m, the normalized matrix is such as Formula (34). Calculate the Euclidean distance between the sample matrix D and the training sample X. The operation process is as (35) and (36).
  (35) $$D=\left[\begin{array}{cccc} d_{11} & d_{12} & \cdots & d_{1m}\\ d_{21} & d_{22} & \cdots & d_{2m}\\ \vdots & \vdots & \ddots & \vdots \\ d_{p1} & d_{p2} & \cdots & d_{pm} \end{array}\right]$$
  (36) $$\begin{array}{l} E=\left[\begin{array}{cccc} \sqrt{\sum_{k=1}^{n}{\left|d_{1k}-c_{1k}\right|}^{2}} & \sqrt{\sum_{k=1}^{n}{\left|d_{1k}-c_{2k}\right|}^{2}} & \cdots & \sqrt{\sum_{k=1}^{n}{\left|d_{1k}-c_{mk}\right|}^{2}}\\ \sqrt{\sum_{k=1}^{n}{\left|d_{2k}-c_{1k}\right|}^{2}} & \sqrt{\sum_{k=1}^{n}{\left|d_{2k}-c_{2k}\right|}^{2}} & \cdots & \sqrt{\sum_{k=1}^{n}{\left|d_{2k}-c_{mk}\right|}^{2}}\\ \vdots & \vdots & \ddots & \vdots \\ \sqrt{\sum_{k=1}^{n}{\left|d_{pk}-c_{1k}\right|}^{2}} & \sqrt{\sum_{k=1}^{n}{\left|d_{pk}-c_{2k}\right|}^{2}} & \cdots & \sqrt{\sum_{k=1}^{n}{\left|d_{pk}-c_{mk}\right|}^{2}} \end{array}\right]\\ =\left[\begin{array}{cccc} E_{11} & E_{12} & \cdots & E_{1m}\\ E_{21} & E_{22} & \cdots & E_{2m}\\ \vdots & \vdots & \ddots & \vdots \\ E_{p1} & E_{p2} & \cdots & E_{pm} \end{array}\right] \end{array}$$
(3) Utilizing the radial basis function as the activation function. The normalized sample to be identified and the training sample are activated to obtain the initial probability matrix P.
  (37) $$P=\left[\begin{array}{cccc} e\frac{-E_{11}}{2\sigma^{2}} & e\frac{-E_{12}}{2\sigma^{2}} & \cdots & e\frac{-E_{1m}}{2\sigma^{2}}\\ e\frac{-E_{21}}{2\sigma^{2}} & e\frac{-E_{22}}{2\sigma^{2}} & \cdots & e\frac{-E_{2m}}{2\sigma^{2}}\\ \vdots & \vdots & \ddots & \vdots \\ e\frac{-E_{p1}}{2\sigma^{2}} & e\frac{-E_{p2}}{2\sigma^{2}} & \cdots & e\frac{-E_{pm}}{2\sigma^{2}} \end{array}\right]=\left[\begin{array}{cccc} P_{11} & P_{12} & \cdots & P_{1m}\\ P_{21} & P_{22} & \cdots & P_{2m}\\ \vdots & \vdots & \ddots & \vdots \\ P_{p1} & P_{p2} & \cdots & P_{pm} \end{array}\right]$$
(4) After the above steps, the output value of the mode layer is calculated. According to (37), the initial probability sum of which fault type belongs to the identified sample in the probabilistic neural network is calculated. The number of fault types representing the training samples, each of which is k.
  (38) $$S=\left[\begin{array}{cccc} \sum_{l=1}^{k}P_{1l} & \sum_{l=2}^{2k}P_{1l} & \cdots & \sum_{l=m-k+1}^{m}P_{1l}\\ \sum_{l=1}^{k}P_{2l} & \sum_{l=2}^{2k}P_{2l} & \cdots & \sum_{l=m-k+1}^{m}P_{2l}\\ \vdots & \vdots & \ddots & \vdots \\ \sum_{l=1}^{k}P_{pl} & \sum_{l=1}^{2k}P_{pl} & \cdots & \sum_{l=m-k+1}^{m}P_{pl} \end{array}\right]=\left[\begin{array}{cccc} S_{11} & S_{12} & \cdots & S_{1c}\\ S_{21} & S_{22} & \cdots & S_{2c}\\ \vdots & \vdots & \ddots & \vdots \\ S_{p1} & S_{p2} & \cdots & S_{pc} \end{array}\right]$$
(5) According to the sum of the initial probability, a maximum probability of the i-th sample to be identified to class j could be calculated. PNN is a classification network model which employs training samples to calculate the maximum estimated probability. For probabilistic neural networks, when the training sample is known, that is, the number of neurons in the pattern layer is determined, once the smoothing factor σ is determined, the parameters and structure of the PNN network are also determined. Therefore, improving the ability of probabilistic neural network fault identification could optimize the parameters of the smoothing factor of the PNN network.

### 4.3. Improved Coyote Optimization Algorithm Based on Probabilistic Neural Network (ICOA-PNN)

In order to avoid the greedy algorithm from falling into the local optimal solution, the improved method proposed in this paper optimizes the iterative greedy algorithm in the growth of the coyotes in the group in the traditional coyote optimization algorithm for the survival of the fittest and the coyote’s life and death two parts, using differential evolution algorithm Substitute. In order to improve the operability of the traditional coyote optimization algorithm, this paper uses the dynamic adjustment of the coyote within the group to replace the coyote in the traditional coyote optimization algorithm. The ICOA consists of two steps: parameter initialization, coyote swarm and coyote growth. 

Step 1: Set parameter initialization and random initialization of coyote groups. Set parameters, such as coyote group size N, coyote group number $N_{p}$, the coyotes’ number in each group $N_{c}$ and MaxDT, where $N=N_{c}\times N_{p}$. Then initialize the coyote group randomly. The randomization operation of j-th dimension of c-th coyote in p-th group is described below. Finally, the social fitness value fit of each coyote soc is calculated, see another formula.

(39) $$sco_{c,j}=lb_{j}+r\times \left(ub_{j}-lb_{j}\right)$$

(40) $$fit_{c}=f\left(soc_{c}\right)$$

where $lb_{j}$ and $ub_{j}$ represent the lower and upper bound of the coyotes’ j-th dimensional social state factor. j=1,2,⋯,D. D is search space dimension. r is a random number uniformly distributed in [0,1]. f is adaptation function. 

Step 2: Effects of the optimal coyote alpha, group culture trends cult and two randomly selected coyotes $cr_{1}$, $cr_{2}$ on coyotes growth. That is, the growth of coyotes in the group is affected by $\sigma_{1}$ and $\sigma_{2}$. Equation (41) is the calculation of cult. The median of all coyotes corresponding to social factors in each factor group (the sequence of social factors after ranking), so cult is also called the median coyote. Equation (42) is the calculation of $\sigma_{1}$ and $\sigma_{2}$. Equation (43) is the growth of coyotes.

(41) $$cult_{j}=\{\begin{array}{c} \begin{array}{cc} O_{\left(N_{c}+1\right)/2,j} & \begin{array}{ccc} N_{c} & is & odd \end{array} \end{array}\\ \begin{array}{cccc} \left(O_{N_{c}/2,j}+O_{\left(N_{c}+1\right)/2,j}\right)/2 & N_{c} & is & even \end{array} \end{array}$$

(42) $$\begin{array}{l} \sigma_{1}=alpha-soc_{cr_{1}}\\ \sigma_{2}=alpha-soc_{cr_{2}}\\ \sigma_{3}=GP-soc_{cr_{1}} \end{array}$$

(43) $$new\_soc_{1}=soc+rn_{1}\times \sigma_{3}+rn_{2}\times \sigma_{2}$$

GP is the current global optimal coyote, which representing the difference between a randomly selected coyote ($cr_{1}$) and GP in the group. $rn_{1}$ and $rn_{2}$ are random numbers generated by a Gaussian (normal) distribution with 0 mean variance. $new\_soc_{1}$ represents new solutions generated by the growth of each coyote within the group under the combined action of $\sigma_{2}$ and $\sigma_{3}$. 

Then the differential evolution is employed to recombine the population evolution according to the differences between individuals to obtain a competitive intermediate population. The offspring and fathers obtain the next generation population through competition and are more competitive. The difference method is as follows:

Step 1: Variation. Select two different individuals $X_{r2}\left(t\right)$ and $X_{r3}\left(t\right)$. Combining them with $X_{r1}\left(t\right)$, the individuals to be mutated after the difference scaling.

(44) $$D_{i}\left(t+1\right)=X_{r1}\left(t\right)+F\times \left(X_{r2}\left(t\right)-X_{r3}\left(t\right)\right)$$

where t is the current number of iterations. F is the mutation operator in [0,2]. $r_{1}$, $r_{2}$, $r_{3}$ are random integers in [1,N] that are not equal to each other and are not i. N is population size. 

Step 2: Cross-cutting. Determining the mutation gene is provided by D(t+1) or X(t+1) by comparing the crossover operator with the random number. The crossover process is as follows.

(45) $$U_{ij}\left(t+1\right)=\{\begin{array}{c} \begin{array}{cccc} \begin{array}{cc} D_{ij}\left(t+1\right) & if \end{array} & -rand\leq CR & or & j=rand\left(1,n\right) \end{array}\\ \begin{array}{cccc} \begin{array}{cc} X_{ij}\left(t+1\right) & if \end{array} & -rand>CR & or & j\neq rand\left(1,n\right) \end{array} \end{array}$$

where CR is a cross operator in [0,1]. rand is a random number in [0,1].

Step 3: Selection. The competition between the middle individual U(t+1) and X(t+1) is obtained by mutation and cross operation. If the parent is superior to the newly acquired offspring, the parent is retained to the next generation, otherwise the offspring is retained to the next generation. The individual selection of coyotes is shown in (46).

(46) $$X_{i}\left(t+1\right)=\{\begin{array}{c} \begin{array}{cc} U_{i}\left(t+1\right) & f\left(U_{i}\left(t+1\right)\right)\leq f\left(X_{i}\left(t\right)\right) \end{array}\\ \begin{array}{cc} X_{i}\left(t\right) & f\left(U_{i}\left(t+1\right)\right)>f\left(X_{i}\left(t\right)\right) \end{array} \end{array}$$

The COA has multiple parameters to be adjusted, which is not easy to operate. Among the improved COA, there are two main parameters, $N_{c}$ and $N_{p}$, which have great influence on the optimization performance. When N is fixed, $N_{p}=N/N_{c}$ if $N_{c}$ is certain. That is, the bigger the $N_{p}$, the smaller the $N_{c}$ and growth operations, while the effect of global solution is enhanced group by group, and mining is strong. In order to improve the operability of the COA, the parameters $N_{p}$ and $N_{c}$ are dynamically adjusted in this paper. Set N=100, then $N_{p}$ and $N_{c}$ should be the factor of 100. According to [25], the number of wolves in each group could not exceed 14, so $N_{c}$ could only be 4,5 and 10. Considering that it takes at least three coyotes to grow, including two randomly selected coyotes and the best coyotes in the group, $N_{c}>3$. When $N_{c}=4$, the optional coyote range is limited, so the most likely values of $N_{c}$ are 5 and 10. The dynamic adjustment parameter scheme is shown in Algorithm 2. The pseudo-code for dynamically adjusting parameters $N_{c}$ and $N_{p}$ is as follow.
**Algorithm 2.** Dynamically adjust parameters1. IF in later period of searching2. $N_{c}=5$3. ELSE in early period of searching4. $N_{c}=10$5. END IF6. $N_{p}=N/N_{c}$ and random grouping

During the later period of searching, $N_{c}=5$, then $N_{p}=20$ The number of groups enhances the positive feedback of the global solution and the local search ability is enhanced. During the early period of searching, the number of groups is small, the positive feedback of global solution is weakened, and the global search ability is enhanced. The dynamic adjustment of coyote numbers’ parameters in the inner suburb of the group not only improves the maneuverability, but also could better balance the exploration and mining ability. In addition, random grouping after dynamic adjustment of parameters could save the coyote group removal and acceptance process, and there is no need to adjust $P_{e}$, which could improve the operability.

As above, a new ICOA-PNN classifier is designed in the paper in view of the advantages of the improved coyote optimization algorithm and PNN. Since both the COA algorithm and the PNN algorithm have good robustness [22,25], the ICOA algorithm proposed in this paper simplifies the process of the COA algorithm without changing its robustness, so the ICOA-PNN method proposed in this paper inherits the above two methods. The advantages of strong robustness also have better robustness. The description is as follows.

(1) Data preprocessing. Data sets are divided into training sets and test sets. The training set and the test set are normalized to [0,1]. Employing the following formula: $v^{\prime }=\left(v-\min\right)/\left(\max-\min\right)$, where v and $v^{\prime }$ represent the original and normalized feature sets respectively. The max and min represent the maximum and minimum eigenvalues respectively.
(2) Initialize the number of coyotes to 100. The maximum number of iterations is 1000. Coyote parameters are selected as random numbers between [−1,1].
(3) Calculate the fitness value of each coyote. To evaluate the quality of each coyote, the average error recognition rate of the training sample is defined as fitness function. This average error recognition rate is achieved by following the triple cross validation process. Given the factors, the PNN parameter optimization problem is formulated as the problem of minimizing fitness functions.
(4) Select the adaptive optimal suburban coyote in the current iteration and consider its location as the current target location T.
(5) Normalize the distance between coyotes to [1,4]. The location of each coyote is updated in each iteration. Update the fitness values for each coyote according to Formula (40). If the updated coyote fitness value is better than the target, the updated coyote will replace the previous coyote. Otherwise, the previous coyotes continue to update.
(6) Determine whether the iterative stop condition is satisfied. If the maximum number of iterations is reached, the loop will terminate and the best target position $c_{best}$ is the output. Otherwise, the algorithm returns to step (2) until the iterative stop condition is satisfied.
(7) Establish the best PNN prediction model by $c_{best}$ and then identify the test data set.

**Figure 5:**
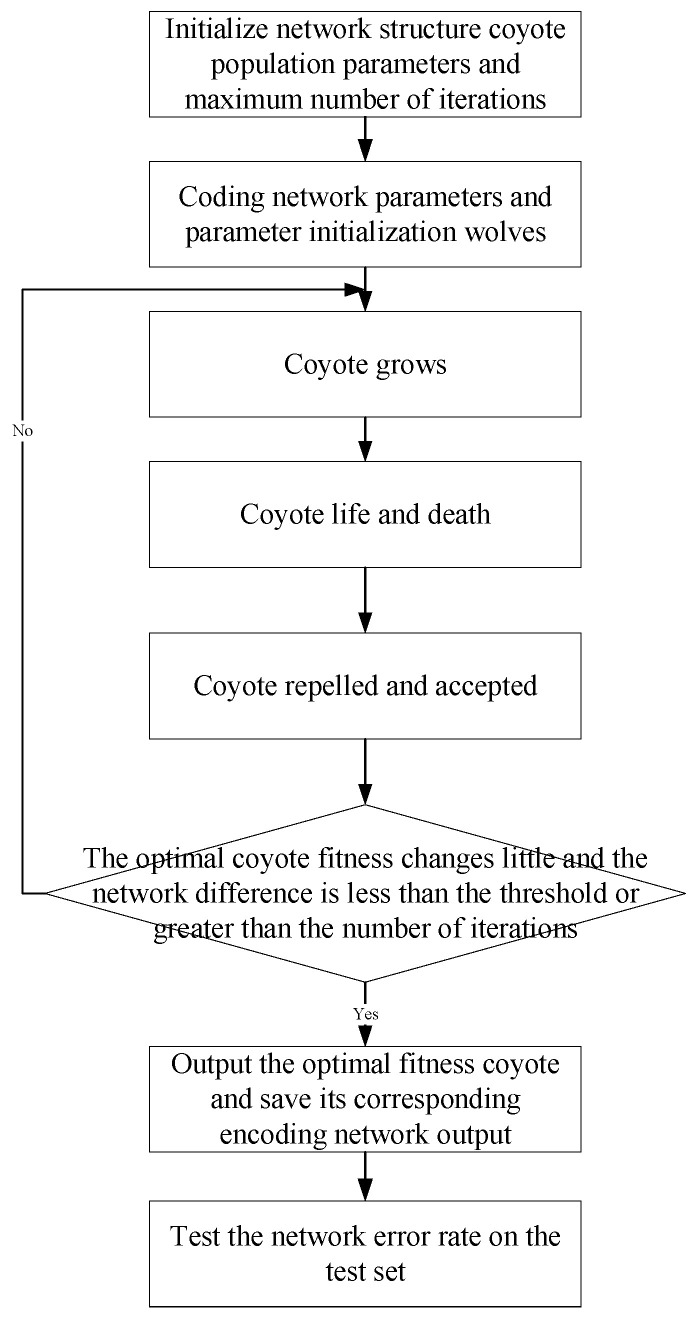
Flow chart of improved coyote optimization algorithm based probabilistic neural network (ICOA-PNN).

The flowchart of ICOA-PNN is shown in Figure 5.

## 5. The Process of Fault Diagnosis

**Figure 6:**
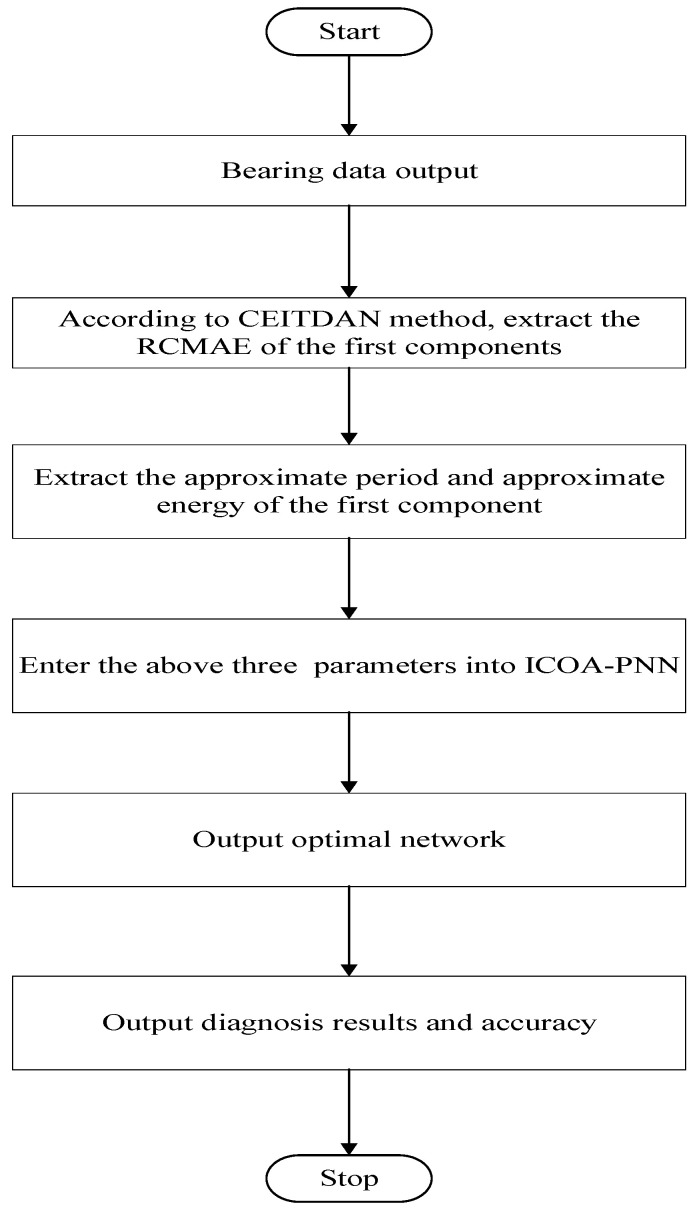
Flow chart of fault diagnosis.

A new rolling bearing fault diagnosis method based on the advantages of RCMAE and approximate period, approximate energy and ICOA-PNN is proposed in this section. Figure 6 is the flow chart of the proposed rolling bearing fault diagnosis method. The process is as follows.

(1) Collect the vibration signal of rolling bearing under different working conditions by acceleration sensor and utilize CEITDAN to decompose it.
(2) Extract the first mode components with the largest correlation coefficient to calculate the RCMAE value. The approximate energy and period of the first mode component are also extracted.
(3) The fault feature set is randomly divided into training sample set and test sample set. The training samples are input into the ICOA-PNN classification for establishing PNN best prediction model. The test samples are entered into the ICOA-PNN prediction model for pattern recognition work.

Due to the decomposition principle of CEITDAN method, the paper chooses the first PRC with the largest correlation coefficient. In the process of decomposing the signal in the CEITDAN method, the mode components are arranged from high frequency to low frequency, and the fault characteristic frequency is generally contained in the mode component with larger correlation coefficient [27], so in order to improve the calculation efficiency, the characteristic parameter dimension is reduced, and then the selection. The first PRC is calculated for RCMAE value, approximate period and approximate energy.

## 6. Experimental Research

Case I: CWRU database fault diagnosis

**Figure 7:**
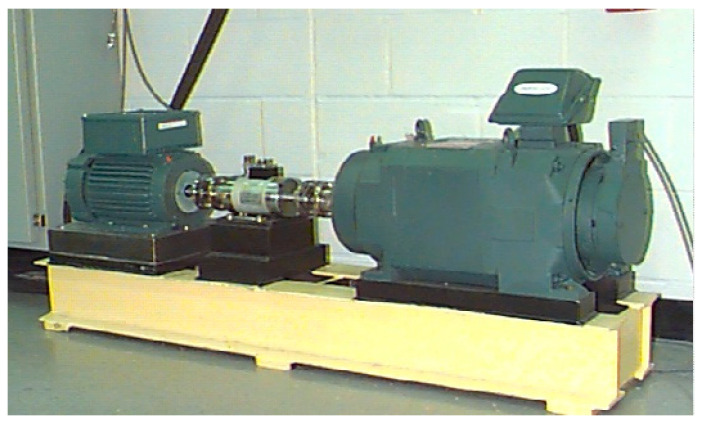
Case Western Reserve University’s experiment platform diagram.

To test the ICOA-PNN performance of the classifier, the experimental data of the rolling bearings are extracted from the CWRU database (see Table 1 for a specific description). The complete experimental platform is shown in Figure 7. The bearing parameters are shown in Table 2. In this experiment, operating conditions of the speed system set to 1797 rpm/min and the sampling frequency is set to 12 K. Firstly, decompose signals by CEITDAN. The first component contains the most important information about vibration. These abnormal faults will cause the sampling data of the system to deviate from the normal noise. The energy of abnormal vibration is also mainly reflected in the main component of the signal.

**Figure 8:**
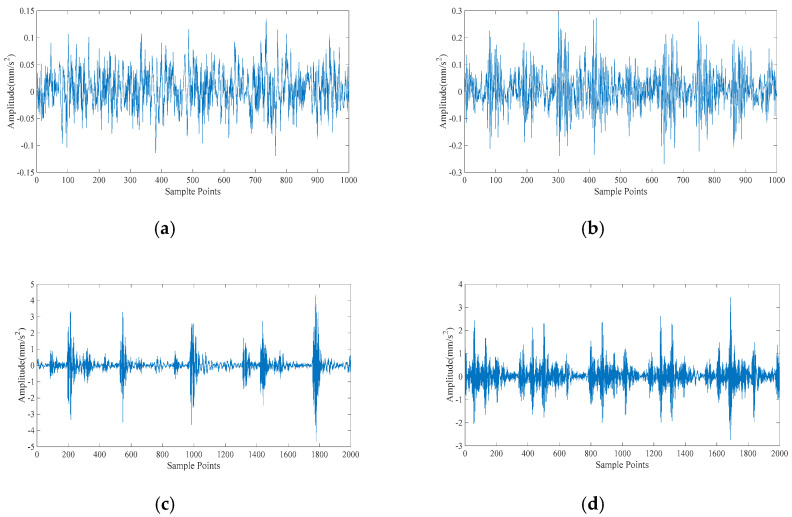

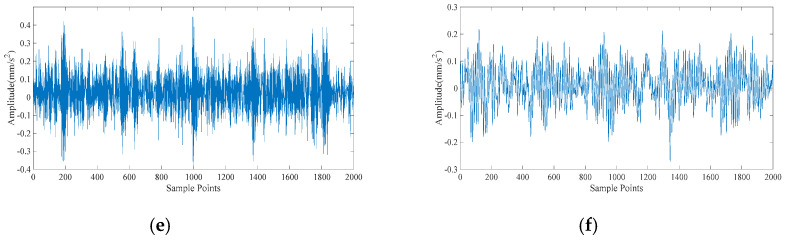
Time domain diagram of rolling bearing under six working conditions: (**a**) is 130 data set; (**b**) is 197 data set; (**c**) is 234 data set; (**d**) is 209 data set; (**e**) is 222 data set; (**f**) is 97 data set.

Each component’s signature features include RCMAE values, vibration period and vibration energy. We choose the approximate energy (i.e., the energy of the abnormal signal) and approximate period (i.e., the frequency of occurrence of the abnormal signal) of the RCMAE value of the first PRC in the CEITDAN decomposition result. For the purpose of verifying the superiority of the proposed optimization PNN method and feature parameter selection, they are compared with PNN, particle swarm optimization based probabilistic neural network (PSO-PNN), firefly algorithm based probabilistic neural network (FA-PNN), chicken swarm optimization based probabilistic neural network (CSO-PNN) and grey wolf optimization based probabilistic neural network (GWO-PNN) respectively. Figure 8 shows the time domain waveforms of six working conditions of rolling bearings. Apparently, it is not easy to distinguish the working state of rolling bearing based on time domain waveform. Given the factors, the proposed method is applied to the fault diagnosis process.

The PNN used in this paper is divided into five layers, and the number of feature input layers is 3. The output classification results are 4 categories: normal, inner ring fault, outer ring fault, rolling body fault. The maximum number of iterations of the coyote algorithm is 1000 and the coyote population is 100. It is randomly divided into 10 groups and the parameters are initialized to random numbers between [−1,1]. In order to verify the superiority of the method proposed in the paper, two kinds of classification are carried out. Firstly, three different outer ring fault sizes are classified, and then the different fault locations and normal states are classified.

**Figure 9:**
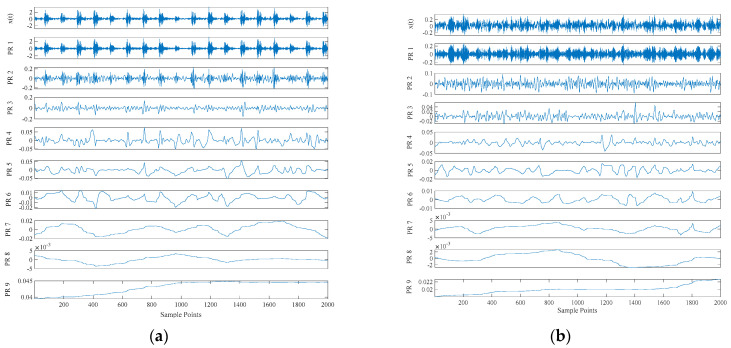

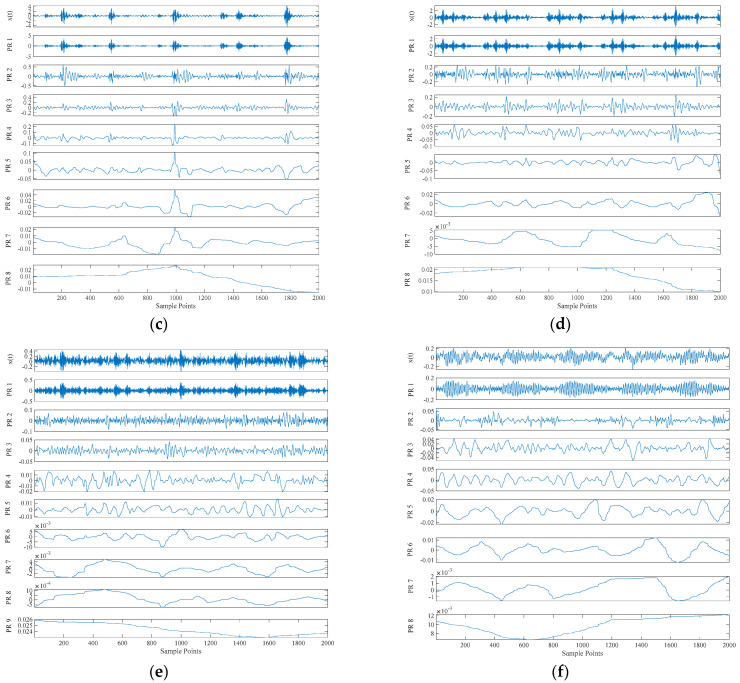
Results of CEITDAN: (**a**) is 130 data set; (**b**) is 197 data set; (**c**) is 234 data set; (**d**) is 209 data set; (**e**) is 222 data set; (**f**) is 97 data set.

**Figure 10:**
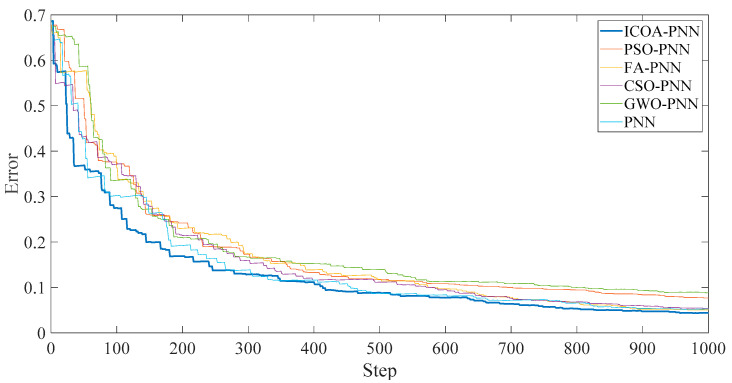
Average error recognition rate of classification methods under different outer ring fault sizes.

In addition, according to Figure 9, the following results can be obtained. Figure 10 shows that the final average fitness value (i.e., average error recognition rate) of ICOA-PNN classifiers under three different outer ring fault sizes is significantly lower than that of other optimized PNN classifiers. It confirms the effectiveness and feasibility of the proposed algorithm for PNN parameter optimization. Firstly, the average recognition accuracy of the PNN classifier based on optimization is significantly higher than that of the original PNN classifier, which indicates that the PNN classifier based on optimization could overcome the problem of parameter selection of the original PNN classifier. Secondly, compared with other PNN classifiers based on optimization, ICOA-PNN classifier has the highest average recognition accuracy for test samples, which verifies its superiority over other PNN classifiers based on optimization.

**Figure 11:**
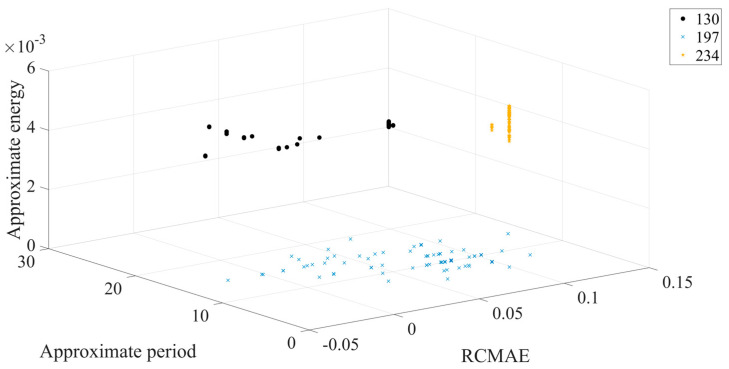
Three-dimensional diagram of classification results of different outer ring fault sizes. (The number name is consistent with the corresponding fault data number in Table 1).

The different colors in Figure 11 represent different outer ring fault sizes, and the outer ring fault sizes correspond to those shown in Table 1. Figure 11 shows that in 3D space, three different outer ring fault sizes are clearly separated from each other, and the aggregation of each working condition is better. Next, the feature set is input to the ICOA-PNN classifier for pattern recognition, and the results are shown in Table 3. It shows that the average recognition accuracy of 3600 test samples is 94.90%.

The above experimental results of rolling bearing fault diagnosis fully confirm the superiority of fault diagnosis methods based on RCMAE and approximate energy, approximate period, and ICOA-PNN. Similarly, it is obvious that the pattern recognition effect of ICOA-PNN is obviously better than that of PNN, PSO-PNN, FA-PNN, CSO-PNN and GWO-PNN classifiers. Through the above classification of different outer ring fault dimensions, the method proposed in this paper can be effectively utilized in bearing fault diagnosis. In order to further verify the effectiveness of this method, the bearing fault data including normal working conditions and three different position faults are classified below.

**Figure 12:**
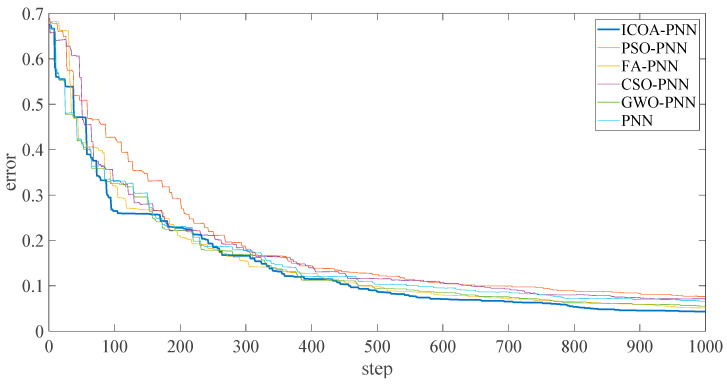
Average error recognition rate of classification methods under different fault types.

Figure 12 shows the final average adaptation value of the ICOA-PNN classifier under different position fault dimensions of three bearings, including normal working conditions (that is, average error recognition rate). It can be found that the average fitness value is significantly lower than other PNN classifiers based on optimization, which confirms the effectiveness and feasibility of the proposed algorithm for PNN parameter optimization. In addition, according to Figure 12, the following results can be obtained. Firstly, the average recognition accuracy of the PNN classifier based on optimization is significantly higher than that of the original PNN classifier, which indicates that the PNN classifier based on optimization could overcome the problem of parameter selection of the original PNN classifier. Secondly, compared with other PNN classifiers based on optimization, ICOA-PNN classifier has the highest average recognition accuracy for test samples, which verifies its superiority over other PNN classifiers based on optimization.

**Figure 13:**
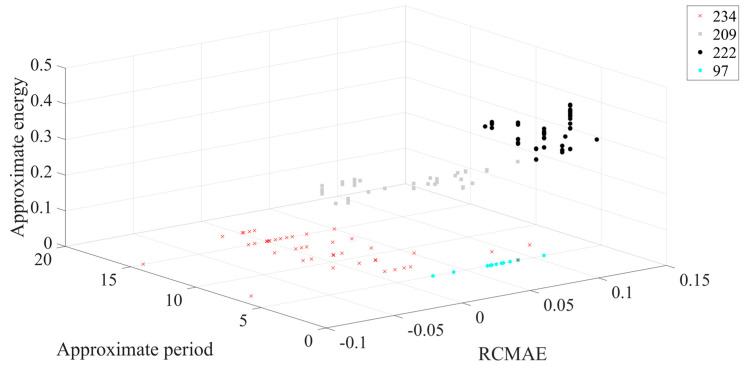
Three-dimensional diagram of classification results of different fault types. (The number name is consistent with the corresponding fault data number in Table 1).

Figure 13 shows that in three-dimensional space, the classification results are obviously separated from each other under four different fault types, and the aggregation of each working condition is better. Next, the feature set is input to the ICOA-PNN classifier for pattern recognition, and the results are shown in Table 4. Table 4 shows that the average recognition accuracy of 3600 test samples is 96.15%.

Case II: Engineering simulation experiment platform fault diagnosis

**Figure 14:**
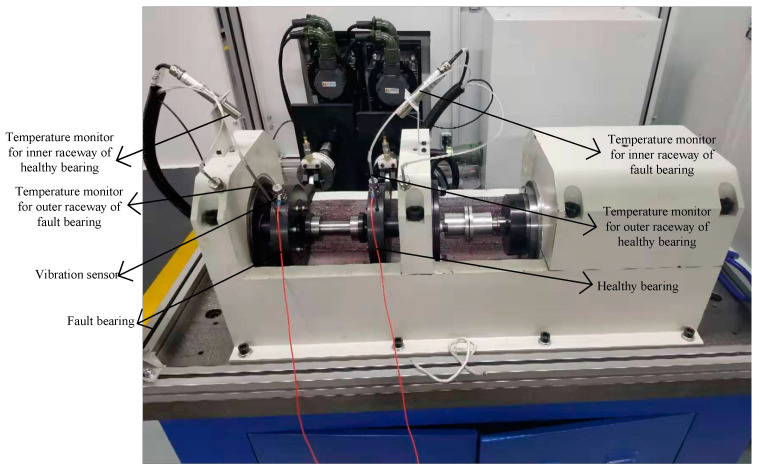
Engineering simulation experiment platform.

The test platform includes two parts: the test platform and the measurement system. The test platform is composed of the test bench and the control system. Figure 14 shows the physical diagram of the test bench, which is mainly composed of the tested bearing, the accompanying bearing, the test spindle, the bearing outer ring fixture, the driving unit and the loading system. During the test, a group of 4 sets of bearings were divided into two sets of tested bearings and two sets of accompanying bearings. The two sets of loading systems load the tested bearings respectively. The driving unit provides power for the whole test bench, and the radial force is provided for the tested bearing by the loading system, which makes the spindle drive the bearing to rotate, and the vibration signal of the bearing in the rotation process is obtained by the vibration sensor. Among them, the driving unit can provide the range of bearing speed is 1000 rpm to 20,000 rpm, and can be adjusted continuously. The loading range of the loading system is that the loading precision can be adjusted continuously. The working limit temperature of the test-bed is 250 °C.

**Figure 15:**
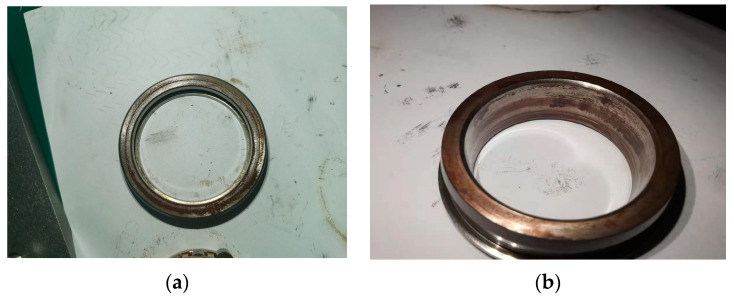
Partial diagram of experimental bearing failure: (**a**) outer ring failure; (**b**) inner ring failure.

The ultimate goal of this paper is to improve the accuracy of bearing fault diagnosis under weak faults under strong background noise. Since the early failures of bearings are generally scratches caused by the friction between the rolling elements and the inner or outer ring, the occurrence of cage and rolling element failures mostly occurs in the middle and late stages, and is accompanied by the characteristic frequency of the inner and outer ring failures, which is a composite failure. So, this experiment only diagnoses the early inner and outer ring faults of the bearing. In order to further verify the method proposed in this article, this part uses the bearing engineering simulation experiment platform built by our laboratory to carry out the experiment. Bearing parameters are shown in Table 5. The failure of the bearing in this experiment is shown in Figure 15.

This part of the experiment is divided into two times, one is the outer ring fault, and the other is the inner ring fault. The sampling frequency was 8192 Hz, and the data length was 4096 points. The bearing rotation speed was 3000 rpm. The CEITDAN method is used for decomposition, and the RCMAE, approximate period and approximate energy of the first rotation component with the largest correlation coefficient are extracted. Two parameters are input into ICOA-PNN method for fault diagnosis.

**Figure 16:**
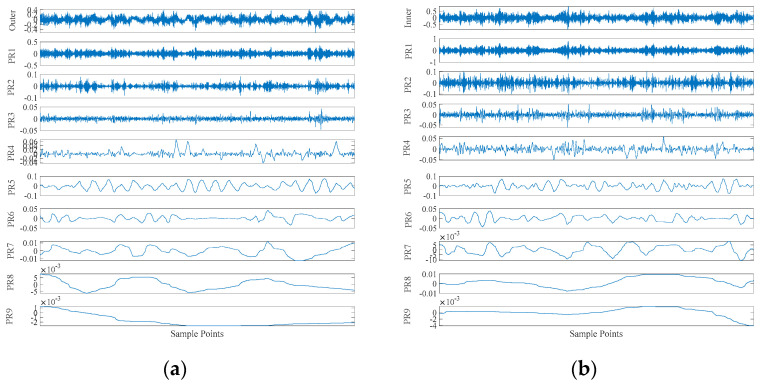
The CEITDAN method decomposes the fault signal diagram of the inner ring and the outer ring: (**a**) outer ring failure; (**b**) inner ring failure.

**Figure 17:**
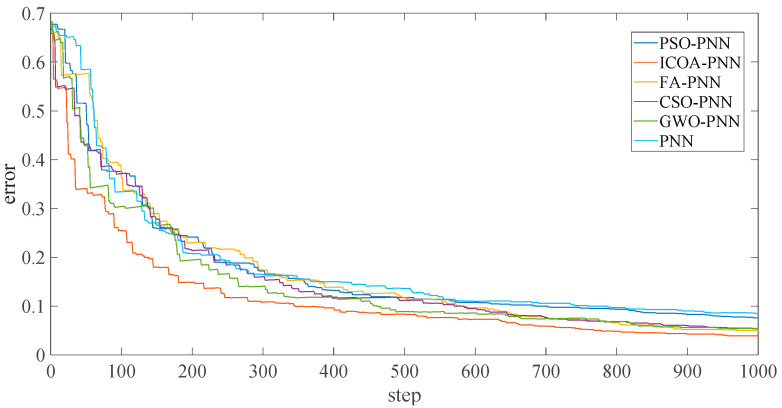
Average error recognition rate of classification methods under different bearing early fault.

In addition, according to Figure 16, the following results can be obtained. As before, this section uses the same comparison criteria. Figure 17 shows that under two different bearing failures, the final average fit value of the ICOA-PNN classifier (i.e., the average error recognition rate) is significantly lower than other optimized PNN classifiers. It is confirmed that the effectiveness and feasibility of the proposed algorithm for PNN parameter optimization are consistent with the previous calculation results of the CWRU database. First, the average recognition accuracy of the optimized PNN classifier is significantly higher than the original PNN classifier, which shows that the optimized PNN classifier can overcome the parameter selection problem of the original PNN classifier. Secondly, compared with other PNN classifiers based on optimization, the ICOA-PNN classifier has the highest average recognition accuracy of test samples, thus proving its superiority over other PNN classifiers based on optimization.

**Figure 18:**
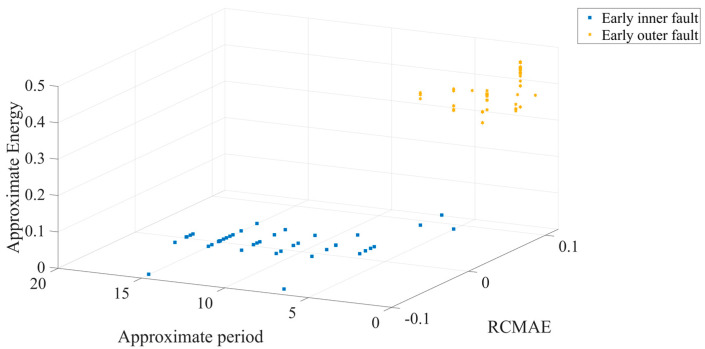
Three-dimensional diagram of classification results of different bearing early fault.

The different colors in Figure 18 represent different bearing faults. Figure 18 shows that in 3D space, two different early failures are clearly separated from each other, and the aggregation of each working condition is better. Next, the feature set is input to the ICOA-PNN classifier for pattern recognition, and the results are shown in Table 6. It shows that the average recognition accuracy of 3600 test samples is 93.9%. Because the early bearing faults are covered in strong background noise, this greatly increases the difficulty of fault diagnosis, which also requires the characteristic parameters to be minimized by noise interference. The method proposed in this paper can effectively improve the accuracy of fault diagnosis, and it performs well in comparison with other algorithms. Other algorithms are all interfered by noise to varying degrees, resulting in a significant decrease in diagnostic accuracy.

## 7. Conclusions

This paper presents a new method for diagnosing rolling bearing early faults. According to the proposed method, RCMAE, approximate period and approximate energy can be used to extract the features of rolling bearing vibration signal, and then the feature set can be input into ICOA-PNN classifier to realize automatic diagnosis of various faults. Based on the experimental data of the rolling bearing, the results show that the method could diagnose the early fault properly and effectively under different working conditions. The study proves that the proposed method is suitable for early fault diagnosis of rolling bearings. For the method proposed in this paper, we could consider improving the optimization algorithm in the future to further improve the optimization of the parameters in the probabilistic neural network, and apply it to the engineering test platform with higher noise intensity.

## Figures and Tables

**Table 1:** Introduction to the six working conditions of rolling bearings.

Data Set Number	Fault Type	Fault Size
130.mat	Outer ring	0.1778 mm
197.mat	Outer ring	0.3556 mm
234.mat	Outer ring	0.5334 mm
209.mat	Inner ring	0.5334 mm
222.mat	Roller	0.5334 mm
97.mat	Normal	-

**Table 2:** Bearing parameters.

Inner Ring Diameter (mm)	Outer Ring Diameter (mm)	Thickness (mm)	Roller Diameter (mm)	Pitch Radius (mm)
25	52	15	7.94	39.04

**Table 3:** Average recognition accuracy based on optimized probabilistic neural network (PNN) and unimproved PNN under different outer ring fault sizes.

Method	Accuracy (%)
ICOA-PNN	94.90
PSO-PNN	93.90
FA-PNN	93.10
CSO-PNN	93.40
GWO-PNN	93.45
PNN	92.55

**Table 4:** Average recognition accuracy based on optimized PNN and unimproved PNN under different fault types.

Method	Accuracy (%)
ICOA-PNN	96.15
PSO-PNN	95.85
FA-PNN	95.10
CSO-PNN	94.75
GWO-PNN	93.80
PNN	95.15

**Table 5:** Bearing parameters.

Ball Number N	Pitch Diameter D	Roller Diameter d	Contact Angle α
14	46	7.5	0

**Table 6:** Average recognition accuracy based on optimized PNN and unimproved PNN under different bearing early fault.

Method	Accuracy (%)
ICOA-PNN	93.90
PSO-PNN	88.90
FA-PNN	88.50
CSO-PNN	87.40
GWO-PNN	88.45
PNN	86.20

## Data Availability

The data included in this study are all owned by the research group and will not be transmitted.
